# Association of anemia and cognitive impairment in patients undergoing maintenance hemodialysis: a cross-sectional study

**DOI:** 10.1186/s12882-025-04336-4

**Published:** 2025-07-15

**Authors:** Lin Huang, Yan Zhang, Jinbao Wang, Hongjin Tang, Jiajun Zhou

**Affiliations:** 1 Blood Purification Center, Affiliated Yijishan Hospital of Wannan Medical College, Wuhu, 241000 PR China; 2 Department of Nephrology, The Second Affiliated Hospital of Anhui Medical University, Hefei, 230601 PR China; 3 College of Biological and Food Engineering, Anhui Polytechnic University, Wuhu, 241000 PR China

**Keywords:** Anemia, Cognitive impairment, Maintenance hemodialysis, Relationship

## Abstract

**Background:**

Cognitive impairment (CI) is common among end-stage renal disease (ESRD) patients undergoing maintenance hemodialysis (MHD), yet its relationship with hemoglobin levels remains underexplored. This study aimed to investigate the association between hemoglobin levels and CI in MHD patients, as well as to identify other contributing factors.

**Methods:**

A cross-sectional study was conducted with 248 MHD patients (49.60% male, mean age 57.23 ± 13.16 years) from a single hemodialysis center. Cognitive function was assessed using the Mini-Mental State Examination (MMSE) with CI defined as a score < 24. Hemoglobin levels were divided into quartiles (Q1: < 90 g/L; Q2: 90–110 g/L; Q3: 110–130 g/L; Q4: >130 g/L). Various independent variables, including age, sex, education level, dialysis duration, comorbidities, and laboratory parameters were analyzed using Spearman correlation test, and univariate and multivariate regression.

**Results:**

Of the 248 patients, 33.90% (84 patients) had CI. Higher hemoglobin quartiles (Q3/Q4) were associated with better cognitive function (higher MMSE scores, *P* < 0.001) and improved performance across cognitive domains. The Spearman and logistic regression analyses revealed the potential associations between cognitive function (MMSE scores) and several factors, including age, education level, dialysis duration, comorbidities, pre-dialysis blood pressure, interdialytic hypotension, albumin, creatinine, uric acid, and hemoglobin (*P* < 0.05). Age (OR = 1.454, *P* < 0.001), male sex (OR = 0.171, *P* = 0.013), pre-dialysis diastolic blood pressure (OR = 0.884, *P* = 0.024), and uric acid (OR = 0.992, *P* = 0.007) were significantly linked with the presence of CI in MHD patients. Multivariate regression further confirmed that adequate hemoglobin concentration was an independent related factor against CI in MHD patients (Model 4, Q1 vs. Q3: OR = 15.395, 95% CI = 3.184–74.443, *P* < 0.001).

**Conclusions:**

Anemia is significantly associated with CI in MHD patients, and can still serve as a clinical marker for early detection and intervention in CI. Maintaining adequate hemoglobin levels may be linked with a reduced CI occurrence in hemodialysis patients. These findings highlight the importance of anemia management and tailored interventions to preserve cognitive function health in ESRD patients.

**Clinical trial number:**

Not applicable.

## Introduction

Chronic kidney disease (CKD) has emerged as a significant global health concern. It is characterized by kidney damage or decreased kidney function lasting more than three months, accompanied by structural or functional abnormalities [1]. The causes of CKD are diverse, including hypertension, hereditary kidney diseases, dyslipidemia, diabetes mellitus, chronic exposure to nephrotoxic agents (like non-steroidal anti-inflammatory drugs), and a number of complications [2]. CKD is generally categorized into five stages based on the glomerular filtration rate, with Stage 5 indicating a glomerular filtration rate below 15 mL/min, leading to end-stage renal disease (ESRD) [3]. Renal replacement therapies, such as kidney transplantation, hemodialysis, and peritoneal dialysis, are crucial treatment options for patients with ESRD. These therapies aid in eliminating excess body fluids and uremic toxins while restoring the body’s homeostasis [4]. In China, about two million individuals suffer from ESRD annually. By 2019, over 630,000 patients were undergoing hemodialysis. Despite the effectiveness of renal replacement therapy, complications commonly arise during hemodialysis. These complications may include insufficient clearance of toxins, cerebral cognitive dysfunction, malnutrition, arterial stiffness, and immune system dysregulation [5]. Addressing these challenges is essential in improving the overall management and outcomes for individuals with ESRD.

Cognitive impairment (CI) refers to the progressive decline in cognitive, sensorimotor, behavioral, and memory functions, leading to disruptions in the daily activities of affected individuals [6]. Clinically, CI is assessed using psychiatric scales such as the Mini-Mental State Examination (MMSE), Montreal Cognitive Assessment (MoCA), and clinical dementia rating [7]. Various factors contribute to CI, including vascular risk factors like hypertension, diabetes, and cardiovascular diseases, deposition of many misfolded proteins (like hyperphosphorylated tau and amyloid-beta), neurodegenerative disorders, and frontotemporal dementia [8]. Cognitive dysfunction is commonly observed in patients with CKD [9]. Particularly, individuals undergoing maintenance hemodialysis (MHD) exhibit a higher incidence of CI compared to those without ESRD. The rising prevalence of cognitive dysfunction in MHD patients impacts both their quality of life and treatment, placing a significant burden on families and society. CI has emerged as a significant predictive factor for all-cause mortality in this population [10, 11]. The reduced cognitive function in MHD patients can be attributed to many factors, such as metabolic abnormalities, old age, cerebrovascular disease, hypertension, dyslipidemia, and malnutrition [12]. Notably, CI and anemia are prevalent among individuals with ESRD. Anemia is considered a significant risk factor, and its treatment has shown promise in improving cognitive function in these patients [13]. Anemia refers to a condition where the concentration of hemoglobin in the blood is below the normal range, leading to a decreased capacity for oxygen transport. According to the World Health Organization (WHO) standards, the normal hemoglobin concentration for adult males should be greater than 130 g/L, for females greater than 120 g/L, and for pregnant women greater than 110 g/L. For patients undergoing dialysis, due to renal failure, the diagnostic criteria for anemia are usually more lenient, with a hemoglobin concentration of less than 110 g/L being considered anemia, and in some cases, it may even be as low as 100 g/L. It is recommended to maintain Hb levels between 100 and 120 g/L, avoiding Hb levels greater than 130 g/L (which may increase cardiovascular risks). The management of anemia in dialysis patients also needs to consider the appearance of symptoms and whether erythropoiesis-stimulating agents (EPO) are used for treatment (KDOQI Clinical Practice Guidelines for Anemia of CKD). Some studies have reported the association of anemia and CI in MHD patients. Hou’s study demonstrated that anemia was linked to impaired neuronal function in CKD patients, with hemoglobin levels showing a negative correlation with striatal function in patients with ESRD [8]. Anemia may exacerbate this issue by leading to increased generation of reactive oxygen species due to insufficient oxygen supply. Anemia reduces the blood′s oxygen-carrying capacity, leading to decreased brain oxygenation and cerebral blood volume, which in turn exacerbates iron metabolism dysregulation and oxidative stress in the brain, resulting in neurodegenerative changes [14, 15].

Although some studies have focused on the relationship between anemia and CI in MHD patients, there is rarely seen in the study on the hemodialysis patients in East China. Moreover, limited research has conducted a systematic and refined subgroup analysis of hemoglobin levels, categorizing them into quartiles and examining their statistical correlation with CI in hemodialysis patients. Therefore, this study aims to investigate the association between anemia and CI in patients undergoing MHD at a single hemodialysis center in East China. This research can provide valuable insights into the impact of anemia on cognitive function in MHD patients, contributing to a better understanding of this critical issue.

## Patients and methods

### Study subjects

This study employed a cross-sectional design to investigate the relationships between hemoglobin levels, biochemical parameters, and CI in hemodialysis patients. A total of 248 ESRD patients, comprising 123 males and 125 females, who had been undergoing MHD for more than 6 months from March 2024 to August 2024, were recruited from the Blood Purification Center of Wannan Medical College Affiliated Yijishan Hospital. The inclusion criteria were as follows: (1) ESRD patients on regular hemodialysis treatment; (2) receiving hemodialysis for more than 3 months, with dialysis sessions lasting 10–12 h per week in a stable condition; (3) age over 18 years. Exclusion criteria included: (1) irregular or inadequate dialysis; (2) presence of dementia or significant concurrent illnesses such as cardiovascular, cerebrovascular, or malignant diseases in the three months prior to the study; (3) communication barriers or unwillingness to complete the questionnaire; (4) immobility, blindness, or deaf-muteness. Detailed individual information, including sociodemographic data (age, gender, years of education), primary cause of kidney failure (hypertension, diabetes, and cardiovascular and cerebrovascular diseases), pre-dialysis systolic blood pressure (Pre-SBP), pre-dialysis diastolic blood pressure (Pre-DBP), interdialytic hypotension, and other relevant clinical data, were collected from each patient. The study received approval from the Ethics Committee of Wannan Medical College Affiliated Yijishan Hospital (Approved number. 20240326), and written informed consent was obtained from all participants involved in the study.

### Hemodialysis method

All the ESRD patients underwent regular hemodialysis sessions three times a week, each lasting 4 h, using a “Gambro” hemodialysis machine and “Gambro” Polyflux L capillary dialyzers. The hemodialysis procedure was conducted through an internal arteriovenous fistula, including native arteriovenous fistulas (89% of the total patients), long-term central venous catheters (6%), and grafted arteriovenous fistulas (5%). Bicarbonate dialysate was used during the dialysis sessions. The blood flow rates ranged from 200 to 260 mL/min, with a constant dialysate flow rate of 500 mL/min. The composition of the dialysate solution included sodium at 138 mmol/L, potassium at 2.0 mmol/L, calcium at 1.50 mmol/L, magnesium at 0.5 mmol/L, chloride at 109.5 mmol/L, and bicarbonate at 32 mmol/L. These specific concentrations were maintained throughout the hemodialysis treatment to ensure proper electrolyte balance and waste removal from the patient’s blood. Erythropoiesis-stimulating agents (ESA), such as erythropoietin or roxadustat, were administered to anemic patients. The dosage was dynamically adjusted based on the patient’s anemia status to ensure optimal management.

### Blood sample measurements

Hemoglobin levels and other biochemical parameters, including albumin, were measured at a single time point for all participants during the study. Hemoglobin levels were assessed immediately prior to the dialysis session, with values obtained from the pre-dialysis blood sample. Biochemical parameters were measured using standardized laboratory techniques. To ensure consistency, all measurements were conducted on the same day within the study period. Only one measurement was recorded per participant, with no repeated assessments over time. Blood samples were obtained from all patients via the antecubital vein after a fasting period of over 8 h. These serum samples were utilized to assess various biochemical parameters, including 25-hydroxyvitamin D (25(OH)D), albumin levels, renal function markers (blood urea nitrogen, serum creatinine, and uric acid), lipid profiles (total cholesterol, triglycerides, high-density lipoprotein cholesterol (HDL-C), and low-density lipoprotein cholesterol (LDL-C)), electrolyte levels (sodium, potassium, calcium, phosphorus, and calcium-phosphorus product), and intact parathyroid hormone (iPTH).

### Cognitive function assessment

Cognitive function assessment is conducted using the Chinese-translated version of the MMSE test, including five parts, namely, orientation, memory, attention and calculation, recall, and language. This assessment evaluates cognitive domains such as naming, orientation, memory, executive function, attention, language ability, and visuospatial skills. The MMSE test, which typically takes around 10 min to administer, is performed by trained physicians either before dialysis or within 1 h after dialysis. The total score of the MMSE test ranges from 0 to 30, with a score less than 24 indicating CI (a commonly accepted threshold) and a score equal to or more than 24 indicating normal cognitive function [16]. Generally, higher MMSE scores correlate with better cognitive function in patients.

### Statistical analysis

Statistical analyses were carried out using SPSS statistical software version 27.0 (IBM Corporation, Armonk, NY, USA). For quantitative data that meet the conditions of normal distribution and homogeneity of variance, the mean ± standard deviation ($\bar {x}$ ± s) was used. Independent samples *t*-test was used for comparison between two groups, and one-way ANOVA was used for comparison among multiple groups. For data with non-normal distribution, median (*P*_*25*_, *P*_*75*_) was used, and Mann-Whitney U test was applied for comparison between two groups, while Kruskal-Wallis *H* test was used for multiple group comparisons. For categorical variables (count data), frequency and percentage were used, and Chi-square test or Fisher’s exact probability test was employed for comparison between groups. The Spearman rank correlation test was used to explore the correlation between the MMSE scores and its sub-scores with various influencing factors (demographic characteristics and biochemical measurements). Based on the quartiles of hemoglobin, patients were divided into four groups: hemoglobin Q1/Q2/Q3/Q4. Univariate logistic regression analysis was conducted to explore the potential factors influencing CI in MHD patients. Further, multivariate logistic regression models were used to analyze the relationship between hemoglobin levels and the presence of CI. A significance level of *P* < 0.05 was considered statistically significant.

## Results

**Fig. 1:**
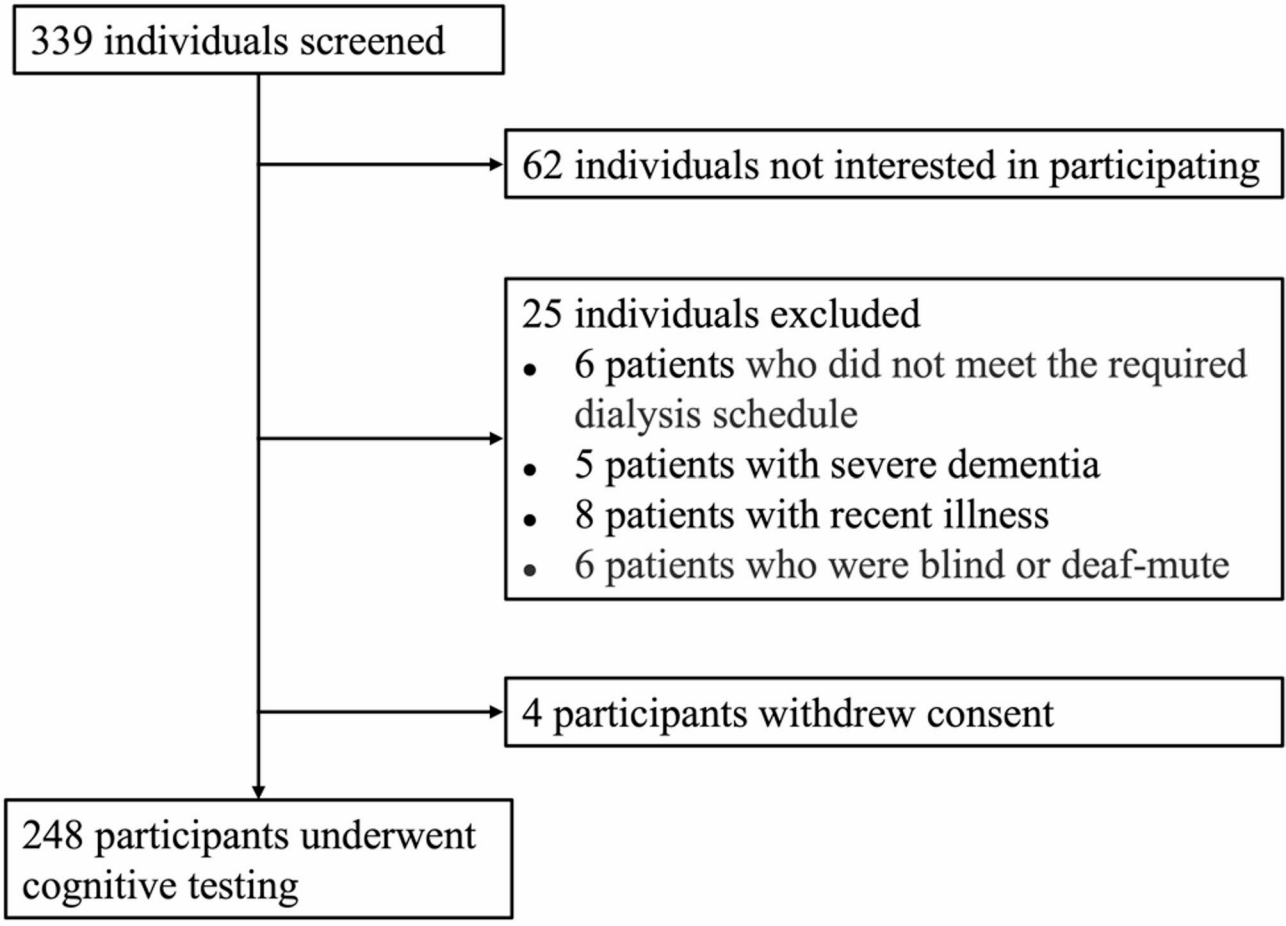
Flow diagram of participant screening. A total of 339 individuals were screened for study eligibility. Of these, 62 individuals were not interested in participation, 25 individuals were excluded based on entry criteria, and 4 individuals withdrew consent, resulting in 248 participants who completed cognitive testing

A total of 339 individuals undergoing maintenance hemodialysis were screened for eligibility to participate in the study (Fig. 1). Of these, 25 individuals were excluded due to failing to meet the inclusion criteria, which included non-adherence to the required dialysis schedule (*n* = 6), severe dementia (*n* = 5), recent illness (*n* = 8), and blindness or being deaf-mute (*n* = 6). Additionally, 62 individuals met the entry criteria but declined to participate in the study. As a result, 252 participants provided initial consent to participate. However, four participants withdrew their consent prior to cognitive testing, leaving 248 participants who subsequently underwent full cognitive assessment.

The demographic information, clinical laboratory data, and MMSE scores of all patients in this study were presented in Table 1. In total, 248 patients receiving MHD were enrolled to this research, including 123 males (49.60%) and 125 females (50.40%). The average age of patients was 57.23 ± 13.16 years old ranging from 23 to 89 years old. The majority of participants had hypertension (80.20%), while diabetes and cardiovascular or cerebrovascular diseases were reported in 24.20% and 20.60% of patients, respectively. The average duration of hemodialysis had a median of 60.00 (24.00, 108.00) months. The clinical laboratory characteristics revealed a mean blood urea nitrogen (BUN) level of 20.64 ± 8.30 mmol/L, with elevated creatinine levels (841.87 ± 304.18 µmol/L) and uric acid (415.15 ± 123.22 µmol/L). The average hemoglobin level was 110.37 ± 26.77 g/L with the value ranging from 43 to 168 g/L. It should be pointed out that the patient with a hemoglobin level of 43 g/L was attributed to erythropoietin resistance upon reviewing the patient’s medical records. The patient with a hemoglobin level of 168 g/L was attributed to the patient’s ongoing treatment with ESA. The use of ESA in hemodialysis patients is common practice to manage anemia. Despite the abnormal Hb levels, these patients were carefully monitored, and no adverse clinical events were reported as a result of the elevated Hb level. All the patients were stable at the time of inclusion in the study, having no acute or life-threatening symptoms. The MMSE scores had a median of 26.00 (23.00–29.00) and 33.90% of the participants (*n* = 84, with score below 24) were identified as CI patients. The subcategories of the MMSE, including the median of orientation 10.00 (10.00, 10.00), memory 3.00 (3.00, 3.00), attention and calculation 4.00 (1.00, 5.00), recall 0.00 (0.00, 2.00), and language 9.00 (9.00, 9.00), were also measured, reflecting cognitive performance across various domains.

**Table 1:** Demographic information, clinical laboratory data, and MMSE score of MHD patients

Variable	Outcome
Age (years)	57.23 ± 13.16
Males, N (%)	123 (49.60%)
Females, N (%)	125 (50.40%)
*Education*,* (years) N (%)*	
0–3	52 (21.00%)
3–6	67 (27.00%)
6–12	111 (44.80%)
> 12	18 (7.20%)
*Antecedents*,* N (%)*	
Hypertension	199 (80.20%)
Diabetes	60 (24.20%)
Cardiovascular and cerebrovascular diseases	51 (20.60%)
Duration of hemodialysis (months)	60.00 (24.00, 108.00)
Pre-SBP (mmHg)	135.00 (128.00, 143.75)
Pre-DBP (mmHg)	74.00 (68.00, 79.00)
Interdialytic hypotension, N (%)	64 (25.81%)
*Clinical laboratory characteristics*	
25(OH)D (ng/mL)	28.64 (22.27, 36.51)
Albumin (g/L)	39.90 (37.43, 42.60)
Blood urea nitrogen (mmol/L)	20.64 ± 8.30
Creatinine (µmol/L)	841.87 ± 304.18
Uric acid (µmol/L)	415.15 ± 123.22
Sodium (mmol/L)	138.26 ± 2.72
Potassium (mmol/L)	4.89 ± 0.93
Calcium (mmol/L)	2.28 (2.13, 2.42)
Phosphorus (mmol/L)	1.77 (1.49, 2.12)
Calcium-phosphorus product (mmol^2^/L^2^)	50.67 (40.75, 61.70)
Total cholesterol (mmol/L)	3.91 (3.19, 4.67)
Triglyceride (mmol/L)	1.44 (1.02, 2.18)
HDL-C (mmol/L)	0.99 (0.84, 1.15)
LDL-C (mmol/L)	2.14 (1.64, 2.57)
iPTH (pg/mL)	244.90 (100.68, 419.45)
Hemoglobin (g/L)	110.37 ± 26.77
MMSE score	26.00 (23.00–29.00)
Orientation	10.00 (10.00, 10.00)
Memory	3.00 (3.00, 3.00)
Attention and Calculation	4.00 (1.00, 5.00)
Recall	0.00 (0.00, 2.00)
Language	9.00 (9.00, 9.00)
MMSE score < 24, N (%)	84 (33.90%)

The demographic information, biochemical characteristics, and MMSE score of all the MHD patients grouped by hemoglobin quartiles were summarized in Table 2. According to hemoglobin quartiles, all patients were divided into four categories: Q1 (< 90 g/L), Q2 (90–110 g/L), Q3 (110–130 g/L), and Q4 (> 130 g/L). The data on age, sex, education level, comorbidities, and other clinical variables were analyzed for differences between the groups. There were no significant differences in age, gender distribution, education level, and interdialytic hypotension across the groups (*P* > 0.05). However, the duration of hemodialysis varied significantly between groups, with a longer duration observed in the higher hemoglobin categories (*P* < 0.001). Regarding biochemical variables, albumin levels were significantly higher in the higher hemoglobin groups (*P* < 0.001), while serum uric acid levels also showed a significant increase in the higher hemoglobin categories (*P* = 0.004). No significant differences were observed for others biochemical characteristics between the groups (*P* > 0.05), such as 25(OH)D, sodium, potassium, phosphorus, and calcium levels. For the MMSE scores, significant differences were observed across all domains, including total MMSE score, orientation, memory, attention and calculation, recall, and language (*P* < 0.001 for most domains, except for language, which had a *P* value of 0.013). The MMSE scores increased progressively from Q1 to Q4, indicating better cognitive performance with relatively higher hemoglobin levels. The attention and calculation domain showed the most pronounced improvement, while the language domain showed a more modest difference across the groups. The number of patients with CI significantly decreased from 37 in Q1 to 8 in Q4 (*P* < 0.001).

**Table 2:** Clinical, biochemical characteristics, and MMSE scores of different groups

Variable	Q1 (*n* = 61)	Q2 (*n* = 63)	Q3 (*n* = 62)	Q4 (*n* = 62)	Statistical value	*P* value
Hemoglobin (g/L)	< 90	90–110	110–130	> 130	–	–
Age (years)	57.92 ± 14.79	57.10 ± 11.91	55.45 ± 13.25	58.47 ± 12.69	0.615	0.606
Males, N (%)	27 (44.30%)	27 (42.90%)	30 (48.40%)	39 (62.90%)	3.146	0.370
Females, N (%)	34 (55.70%)	36 (57.10%)	32 (51.60%)	23 (37.10%)	3.160	0.368
*Education (years)*						
0–3	20 (32.80%)	13 (20.60%)	9 (14.50%)	10 (16.10%)	5.692	0.128
3–6	20 (32.80%)	18 (28.60%)	17 (27.40%)	12 (19.40%)	2.075	0.557
6–12	16 (26.20%)	30 (47.60%)	30 (48.40%)	35 (56.50%)	7.234	0.065
> 12	5 (8.20%)	2 (3.20%)	6 (9.70%)	5 (8.10%)	2.000	0.572
*Antecedents*,* N (%)*						
Hypertension	55 (90.20%)	45 (71.40%)	48 (77.40%)	51 (82.30%)	1.101	0.777
Diabetes	22 (36.10%)	16 (25.40%)	13 (21.00%)	9 (14.50%)	6.000	0.112
Cardiovascular and cerebrovascular diseases	16 (26.20%)	16 (25.40%)	13 (21.00%)	6 (9.70%)	5.235	0.155
Duration of hemodialysis (months)	24.00 (12.00, 78.00)	60.00 (24.00, 108.00)	48.00 (24.00, 108.00)	96.00 (36.00, 144.00)	17.595	< 0.001
Pre-SBP (mmHg)	136.00 (127.00, 147.50)	133.00 (128.00, 140.00)	132.50 (127.75, 140.50)	136.00 (129.50, 141.00)	1.163	0.762
Pre-DBP (mmHg)	75.00 (68.00, 79.00)	76.00 (70.00, 82.00)	71.00 (66.00, 78.00)	71.50 (67.75, 78.00)	5.756	0.124
Interdialytic hypotension, N (%)	25 (39.10%)	17 (25.70%)	14 (21.90%)	8 (12.50%)	5.264	0.135
*Clinical laboratory characteristics*						
25(OH)D (ng/mL)	27.89 (19.94, 33.76)	28.98 (23.82, 38.12)	27.61 (22.68, 36.85)	29.76 (21.21, 38.56)	2.609	0.456
Albumin (g/L)	37.80 (33.25, 39.95)	40.40 (38.30, 42.40)	40.25 (38.68, 43.73)	40.85 (37.88, 43.65)	28.961	< 0.001
Blood urea nitrogen (mmol/L)	18.85 ± 8.61	20.64 ± 7.33	22.30 ± 8.57	20.75 ± 8.48	1.794	0.149
Creatinine (µmol/L)	764.09 ± 349.01	847.20 ± 281.44	911.47 ± 273.90	843.37 ± 97.18	2.461	0.063
Uric acid (µmol/L)	370.47 ± 127.55	411.34 ± 132.35	430.57 ± 110.28	447.57 ± 110.39	4.645	0.004
Sodium (mmol/L)	136.72 ± 1.89	137.91 ± 2.73	136.87 ± 2.01	138.03 ± 2.44	2.071	0.388
Potassium (mmol/L)	4.63 ± 4.58	4.88 ± 0.99	4.96 ± 0.76	5.08 ± 1.02	2.620	0.051
Calcium (mmol/L)	2.24 (2.10, 2.42)	2.27 (2.12, 2.40)	2.30 (2.13, 2.42)	2.30 (2.19, 2.43)	1.760	0.624
Phosphorus (mmol/L)	1.74 (1.32, 2.18)	1.77 (1.51, 2.00)	1.86 (1.49, 2.01)	1.82 (1.59, 2.14)	3.085	0.379
Calcium-phosphorus product (mmol^2^/L^2^)	46.71 (34.45, 63.01)	50.93 (41.70, 56.96)	51.15 (40.10, 66.69)	52.11 (44.55, 60.96)	4.045	0.257
Total cholesterol (mmol/L)	3.84 (3.05, 4.36)	3.73 (2.94, 5.04)	4.05 (3.27, 4.75)	4.05 (3.53, 4.76)	5.483	0.140
Triglyceride (mmol/L)	1.25 (0.94, 2.08)	1.47 (1.09, 2.02)	1.53 (1.08, 2.32)	1.48 (1.06, 2.37)	2.488	0.477
HDL-C (mmol/L)	0.96 (0.82, 1.13)	0.97 (0.83, 1.14)	1.01 (0.86, 1.10)	1.04 (0.90, 1.27)	3.404	0.333
LDL-C (mmol/L)	2.21 (1.84, 2.63)	1.89 (1.30, 2.31)	2.31 (1.63, 2.56)	2.14 (1.64, 2.68)	11.584	0.009
iPTH (pg/mL)	246.70 (100.55, 598.62)	249.70 (59.50, 417.50)	240.20 (132.10, 415.93)	240.00 (88.35, 400.63)	1.191	0.755
Hemoglobin (g/L)	75.56 ± 10.99	100.29 ± 6.15	120.66 ± 5.82	144.58 ± 9.87	737.042	< 0.001
MMSE score	23.00 (20.00, 25.00)	24.00 (22.00, 26.00)	28.00 (25.75, 29.00)	30.00 (28.00, 30.00)	85.220	< 0.001
Orientation	10.00 (8.00, 10.00)	10.00 (9.00, 10.00)	10.00 (10.00, 10.00)	10.00 (10.00, 10.00)	31.973	< 0.001
Memory	3.00 (2.50, 3.00)	3.00 (3.00, 3.00)	3.00 (3.00, 3.00)	3.00 (3.00, 3.00)	19.648	< 0.001
Attention and Calculation	1.00 (0.00, 3.00)	2.00 (1.00, 4.00)	5.00 (4.00, 5.00)	5.00 (5.00, 5.00)	83.060	< 0.001
Recall	0.00 (0.00, 0.00)	0.00 (0.00, 1.00)	1.00 (0.00, 2.00)	3.00 (1.00, 3.00)	82.233	< 0.001
Language	9.00 (9.00, 9.00)	9.00 (9.00, 9.00)	9.00 (9.00, 9.00)	9.00 (9.00, 9.00)	10.828	0.013
CI, MMSE score < 24, N (%)	37 (60.70%)	26 (41.30%)	13 (21.00%)	8 (12.90%)	37.856	< 0.001

Furthermore, the correlation between MMSE score and the demographic and clinical laboratory parameters of the participants was analyzed using the Spearman rank correlation test (Table 3). Age showed a strong negative correlation with the MMSE score across all domains (ρ = − 0.455 to − 0.708, *P* < 0.001), indicating that older age was associated with lower cognitive function. Sex was not significantly correlated with the MMSE score, except in the memory domain (ρ = 0.153, *P* = 0.016). Education exhibited a positive correlation with MMSE scores across all domains (ρ = 0.431 to 0.763, *P* < 0.001). Several medical conditions, including hypertension, diabetes, cardiovascular diseases, and interdialytic hypotension, demonstrated negative correlations with cognitive performance, particularly in orientation, memory, and recall domains (ρ = − 0.250 to − 0.294, *P* < 0.001). The duration of hemodialysis was positively correlated with MMSE scores, especially in orientation and recall domains (ρ = 0.230 to 0.261, *P* < 0.001). Both pre-systolic blood pressure (Pre-SBP) and pre-diastolic blood pressure (Pre-DBP) were negatively correlated with the MMSE score, particularly in memory, recall, and language domains (ρ = − 0.357 to − 0.496, *P* < 0.001). Among laboratory parameters, hemoglobin levels showed a positive correlation with MMSE scores in all domains (ρ = 0.175 to 0.564, *P* < 0.001), as did creatinine levels (*P* < 0.05). No significant correlations were found between 25(OH)D, potassium, sodium, calcium, or phosphorus levels and cognitive performance.

**Table 3:** Correlation analysis between MMSE score and others parameters using spearman rank correlation analysis

Variable	MMSE score		Orientation		Memory		Attention and calculation		Recall		Language	
	ρ	*P* value	ρ	*P* value	ρ	*P* value	ρ	*P* value	ρ	*P* value	ρ	*P* value
Age (years)	–0.708	< 0.001	–0.628	< 0.001	–0.455	< 0.001	–0.662	< 0.001	–0.580	< 0.001	–0.501	< 0.001
Sex (N)	0.108	0.090	0.132	0.038	0.153	0.016	0.090	0.158	0.087	0.173	0.049	0. 442
Education (years)	0.763	< 0.001	0.633	< 0.001	0.431	< 0.001	0.742	< 0.001	0.631	< 0.001	0.467	< 0.001
*Antecedents*,* N (%)*												
Hypertension	0.023	0.714	–0.006	0.923	–0.043	0.505	–0.001	0.988	0.041	0.525	0.065	0.304
Diabetes	–0.280	< 0.001	–0.226	< 0.001	–0.195	0.002	–0.253	< 0.001	–0.250	< 0.001	–0.115	0.070
Cardiovascular and cerebrovascular diseases	–0.275	< 0.001	–0.204	0.001	–0.154	0.015	–0.234	< 0.001	–0.294	< 0.001	–0.152	0.017
Duration of hemodialysis (months)	0.259	< 0.001	0.119	0.062	0.077	0.230	0.261	< 0.001	0.230	< 0.001	0.117	0.065
Pre-SBP (mmHg)	–0.496	< 0.001	–0.456	< 0.001	–0.247	< 0.001	–0.471	< 0.001	–0.410	< 0.001	–0.408	< 0.001
Pre-DBP (mmHg)	–0.469	< 0.001	–0.381	< 0.001	–0.310	< 0.001	–0.403	< 0.001	–0.412	< 0.001	–0.357	< 0.001
Interdialytic hypotension, N (%)	–0.181	< 0.001	–0.204	< 0.001	–0.172	0.005	–0.203	< 0.001	–0.221	< 0.001	–0.137	0.090
*Clinical laboratory characteristics*												
25(OH)D (ng/mL)	–0.064	0.316	0.010	0.871	0.055	0.387	–0.070	0.271	–0.075	0.239	0.009	0.887
Albumin (g/L)	0.273	< 0.001	0.206	0.001	0.069	0.281	0.262	< 0.001	0.252	< 0.001	0.124	0.062
Blood urea nitrogen (mmol/L)	0.091	0.152	0.123	0.053	0.107	0.094	0.102	0.110	0.038	0.552	0.063	0.321
Creatinine (µmol/L)	0.229	< 0.001	0.238	< 0.001	0.171	0.007	0.220	< 0.001	0.180	0.005	0.136	0.033
Uric acid (µmol/L)	0.217	< 0.001	0.159	0.012	0.139	0.029	0.230	< 0.001	0.128	0.044	0.174	0.006
Sodium (mmol/L)	0.176	0.409	0.231	0.320	0.284	0.141	0.092	0.104	0.183	0.097	0.096	0.305
Potassium (mmol/L)	0.090	0.157	0.051	0.420	–0.004	0.955	0.095	0.135	0.084	0.185	–0.019	0.770
Calcium (mmol/L)	0.033	0.601	0.092	0.148	0.028	0.661	0.025	0.696	0.007	0.910	0.071	0.266
Phosphorus (mmol/L)	0.136	0.033	0.155	0.014	0.082	0.197	0.152	0.017	0.104	0.103	0.079	0.214
Calcium-phosphorus product (mmol^2^/L^2^)	0.129	0.042	0.170	0.007	0.070	0.271	0.147	0.021	0.092	0.151	0.079	0.217
Total cholesterol (mmol/L)	0.102	0.109	0.062	0.329	0.022	0.732	0.074	0.247	0.130	0.041	0.037	0.559
Triglyceride (mmol/L)	0.133	0.036	0.142	0.025	0.012	0.855	0.111	0.081	0.128	0.043	0.119	0.061
HDL-C (mmol/L)	0.085	0.181	0.005	0.936	0.048	0.447	0.062	0.332	0.103	0.105	–0.024	0.707
LDL-C (mmol/L)	0.003	0.967	0.064	0.317	–0.030	0.636	0.016	0.799	0.008	0.894	–0.006	0.922
iPTH (pg/mL)	–0.013	0.837	0.078	0.221	0.057	0.371	–0.043	0.499	–0.033	0.608	0.062	0.328
Hemoglobin (g/L)	0.564	< 0.001	0.358	< 0.001	0.260	< 0.001	0.555	< 0.001	0.540	< 0.001	0.175	0.006

The logistic regression analysis for both univariate and multivariate models revealed several factors significantly associated with CI in MHD patients (Table 4). All the variables with *P* < 0.05 in univariate models, including age, male, education, antecedents, duration of hemodialysis, Pre-SBP, Pre-DBP, interdialytic hypotension, albumin, creatinine, uric acid, phosphorus, and hemoglobin, were considered and included during the multivariate regression analysis. In the univariate analysis, age was a significant predictor of CI, with an odds ratio (OR) of 1.226 (95% CI: 1.164–1.291, *P* < 0.001). The association remained strong in the multivariate analysis, where the OR was 1.454 (95% CI: 1.227–1.723, *P* < 0.001). Gender also showed a significant association, with male patients having a lower odds of CI compared to females (OR = 0.573, 95% CI: 0.336–0.977, *P* = 0.041 in univariate analysis; OR = 0.171, 95% CI: 0.043–0.687, *P* = 0.013 in multivariate analysis). This suggests that being male may be protective against CI in this patient cohort. Regarding education, the univariate analysis revealed that patients with more education had significantly reduced CI occurrence. Specifically, patients with 3–6 years of education had an OR of 0.025 (95% CI: 0.006–0.113, *P* < 0.001), while those with 6–12 years of education had an OR of 0.003 (95% CI: 0.001–0.015, *P* < 0.001). However, for patients with more than 12 years of education, no significant association was found (OR = 0.000, *P* = 0.998), possibly due to the limited sample amount. Comorbidities, such as diabetes (OR = 2.989, 95% CI: 1.641–5.443, *P* < 0.001), cardiovascular and cerebrovascular diseases (OR = 2.763, 95% CI: 1.471–5.189, *P* = 0.002) were significantly associated with higher CI occurrence in the univariate analysis, as well as interdialytic hypotension (OR = 2.375, 95% CI: 1.407–5.129, *P* < 0.001). Additionally, the duration of hemodialysis showed a significant negative association with the presence of CI, with an OR of 0.991 (95% CI: 0.986–0.996, *P* < 0.001), suggesting that longer dialysis may contribute to reducing CI event. In the clinical laboratory characteristics, albumin levels (OR = 0.900, 95% CI: 0.849–0.955, *P* < 0.001) and hemoglobin levels (OR = 0.968, 95% CI: 0.956–0.979, *P* < 0.001 in univariate analysis and OR = 0.899, 95% CI: 0.861–0.939, *P* < 0.001 in multivariate analysis) were significantly inversely associated with CI. Uric acid levels also showed a significant association with CI occurrence (OR = 0.996, 95% CI: 0.993–0.998, *P* < 0.001 in univariate; OR = 0.992, 95% CI: 0.986–0.998, *P* = 0.007 in multivariate).

**Table 4:** Correlation analysis between clinical, biochemical parameters and CI in MHD patients through logistic regression analysis

Variable	Univariate analysis		Multivariate analysis	
	OR (95% CI)	*P* value	OR (95% CI)	*P* value
Age (years)	1.226 (1.164–1.291)	< 0.001	1.454 (1.227–1.723)	< 0.001
Females	Ref.		Ref.	
Male	0.573 (0.336–0.977)	0.041	0.171 (0.043–0.687)	0.013
*Education (years)*				
0–3	Ref.		Ref.	
3–6	0.025 (0.006–0.113)	< 0.001	0.240 (0.018–3.141)	0.277
6–12	0.003 (0.001–0.015)	< 0.001	0.281 (0.012–6.831)	0.435
> 12	0.000 (0.000–0.000)	0.998	0.000 (0.000–0.000)	0.998
*Antecedents*,* N (%)*				
Hypertension	1.071 (0.551–2.082)	0.841		
Diabetes	2.989 (1.641–5.443)	< 0.001	0.729 (0.191–2.781)	0.644
Cardiovascular and cerebrovascular diseases	2.763 (1.471–5.189)	0.002	2.678 (0.693–10.349)	0.153
Duration of hemodialysis (months)	0.991 (0.986–0.996)	< 0.001	1.002 (0.993–1.012)	0.629
Pre-SBP (mmHg)	1.120 (1.085–1.156)	< 0.001	1.036 (0.958–1.122)	0.375
Pre-DBP (mmHg)	1.108 (1.066–1.150)	< 0.001	0.884 (0.794–0.984)	0.024
Interdialytic hypotension, N (%)	2.375 (1.407–5.129)	< 0.001	2.051 (1.792–4.983)	0.132
*Clinical laboratory characteristics*				
25(OH)D (ng/mL)	1.005 (0.981–1.029)	0.704		
Albumin (g/L)	0.900 (0.849–0.955)	< 0.001	1.036 (0.901–1.190)	0.620
Blood urea nitrogen (mmol/L)	0.976 (0.945–1.008)	0.142		
Creatinine (µmol/L)	0.999 (0.998-1.000)	0.002	1.001 (0.999–1.004)	0.284
Uric acid (µmol/L)	0.996 (0.993–0.998)	< 0.001	0.992 (0.986–0.998)	0.007
Sodium (mmol/L)	0.989 (0.981–0.997)	0.264		
Potassium (mmol/L)	0.895 (0.673–1.189)	0.443		
Calcium (mmol/L)	0.700 (0.231–2.120)	0.528		
Phosphorus (mmol/L)	0.581 (0.353–0.956)	0.033	0.630 (0.227–1.746)	0.374
Calcium-phosphorus product	0.984 (0.969–1.001)	0.058		
Total cholesterol (mmol/L)	0.937 (0.742–1.182)	0.581		
Triglyceride (mmol/L)	0.938 (0.701–1.254)	0.666		
HDL-C (mmol/L)	0.786 (0.353–1.753)	0.557		
LDL-C (mmol/L)	1.015 (0.683–1.508)	0.942		
iPTH (pg/mL)	1.000 (0.999-1.000)	0.579		
Hemoglobin (g/L)	0.968 (0.956–0.979)	< 0.001	0.899 (0.861–0.939)	< 0.001

The association between hemoglobin levels and CI in MHD patients was further analyzed across four models using the Q3 group (normal hemoglobin level) as a reference (Table 5). The results demonstrate a strong and significant relationship between hemoglobin levels and the presence of CI, with varying levels of adjustment across the models. In Model 1 (unadjusted model), the patients in the Q1 group (hemoglobin < 90 g/L) had a significantly higher risk of CI compared to the Q3 group, with an odds ratio (OR) of 5.811 (95% CI: 2.615–12.914, *P* < 0.001). After adjustment for diabetes, cardiovascular and cerebrovascular diseases, Pre-SBP, Pre-DBP, and interdialytic hypotension based on Model 1, the CI occurrence was even higher in subsequent models, with Model 2 showing an OR of 11.248 (95% CI: 3.749–33.751, *P* < 0.001) for Q1. In Model 3 with adjusting blood urea nitrogen, creatinine, uric acid, phosphorus, albumin, and iPTH based on Model 2, the association remained significant, with Q1 showing an OR of 13.445 (95% CI: 3.900-46.345, *P* < 0.001). After further adjustment in Model 4 for age, sex, education, and duration of hemodialysis, the association remained significant (OR = 15.395, 95% CI: 3.184–74.443, *P* < 0.001). For the second hemoglobin quartile (Q2), the presence of CI was also elevated but less pronounced than Q1. In Model 1, the OR was 2.649 (95% CI: 1.201–5.841, *P* = 0.016), and it remained significant in Model 2 and Model 3 (OR = 4.523, 95% CI: 1.564–13.080, *P* = 0.005 and OR = 4.693, 95% CI: 1.559–14.131, *P* = 0.006, respectively). In Model 4, the association enhanced with the OR increasing to 6.085 (95% CI: 1.477–25.066, *P* = 0.012), indicating a potential improvement after adjusting for additional factors. The results for the Q4 group varied across Model 1 to Model 3 and did not show significant changes in CI event, except in Model 4 (OR = 0.005, 95% CI: 0.002–0.042, *P* = 0.021).

**Table 5:** The association between hemoglobin level and CI in MHD patients

No.	Model 1		Model 2		Model 3		Model 4	
	OR (95% CI)	*P* value	OR (95% CI)	*P* value	OR (95% CI)	*P* value	OR (95% CI)	*P* value
Q1: < 90 g/L	5.811 (2.615–12.914)	< 0.001	11.248 (3.749–3.751)	< 0.001	13.445 (3.900-46.345)	< 0.001	15.395 (3.184–74.443)	< 0.001
Q2: 90–110 g/L	2.649 (1.201–5.841)	0.016	4.523 (1.564–13.080)	0.005	4.693 (1.559–14.131)	0.006	6.085 (1.477–25.066)	0.012
Q3: 110–130 g/L	1.00 (Reference)	–	1.00 (Reference)	–	1.00 (Reference)	–	1.00 (Reference)	–
Q4: > 130 g/L	0.558 (0.213–1.461)	0.235	0.400 (0.115–1.391)	0.150	0.400 (0.112–1.432)	0.159	0.005 (0.002–0.042)	0.021

Model 1: Unadjusted model

Model 2: Adjusted diabetes, cardiovascular and cerebrovascular diseases, Pre-SBP, Pre-DBP, and interdialytic hypotension based on Model 1

Model 3: Adjusted blood urea nitrogen, creatinine, uric acid, phosphorus, albumin, and iPTH based on Model 2

Model 4: Adjusted age, sex, education, and duration of hemodialysis based on Model 3

## Discussion

In recent years, the number of CKD patients receiving MHD treatment is gradually increasing. CI can hinder these patients′ comprehension and processing of information, affecting their involvement in healthcare decisions, adherence to complex medical regimens, and proper management of diet and fluid intake [17]. Moreover, CI is linked to increased hospitalization and mortality rates, as well as diminished quality of life [18]. Therefore, cognitive dysfunction poses a significant challenge for those on MHD, which has garnered significant interest. Many potential pathophysiologic derangements have been identified in CI among MHD patients, including elevated uremic toxins, intracranial infarctions, dialysis imbalances, oxidative stress, chronic inflammation, and elevated homocysteine levels [19]. It was reported that 25% of MHD patients within 48–82 years old were cognitively impaired in Laouad′s study focusing on the prevalence and risk factors of cognitive dysfunction in chronic hemodialysis patients [16]. Pulignano et al. reported that 38.9% of 190 patients were diagnosed as CI in older patients aged at least 70 years with heart failure [20]. Li et al. found that 31.5% of 146 ESRD patients undergoing MHD were identified as CI using MoCA test [21]. In our study, the MMSE scores revealed that a substantial proportion (33.90%) of the patients had CI. This aligned with previous study that indicated a high prevalence of cognitive decline in patients undergoing chronic dialysis, possibly due to a combination of factors such as uremia, anemia, and electrolyte imbalances. In terms of the existed difference on the prevalence of CI in MHD patients, it may be attributed to the sample size and their racial differences enrolled in the research. The relationship between anemia and cognitive dysfunction was particularly noteworthy, as the hemoglobin levels in this cohort were relatively low (110.37 ± 26.77 g/L). These findings highlighted that the patients with higher hemoglobin levels (Q3 and Q4) demonstrated better cognitive performance compared to those with lower hemoglobin levels (Q1 and Q2). This was consistent with the well-established link between anemia and cognitive decline, particularly in populations undergoing hemodialysis. Furthermore, the MMSE subcategories revealed specific cognitive deficits in various domains. This is consistent with the hypothesis that CI in dialysis patients may involve executive dysfunction, memory problems, and difficulties with attention and concentration.

Research in the general population indicates that CI may be associated with conventional vascular risk factors such as aging, diabetes, hypertension, dyslipidemia, and smoking, as well as nontraditional factors like elevated homocysteine levels, oxidative stress, and chronic inflammation [22]. Especially for ESRD patients, there is a notable prevalence of both established and potential cerebrovascular risk factors [23]. Previous studies have identified that in the dialysis population, cognitive dysfunction is closely linked to factors beyond the usual risk factors, including blood exposure, vascular endothelial damage, uremic toxin buildup, dyslipidemia, malnutrition, hormone insufficiency, and anemia [24, 25]. Pei et al. also found that multiple risk factors, such as age, education years, serum albumin, blood pressure, comorbidities, and uric acid, were significantly related to mild CI in MHD patients based on the Spearman correlation analysis, influencing its incidence and progression [26]. In our study, these findings demonstrated the relationship between cognitive function and clinical and biochemical factors, such as age, education, chronic medical conditions, and laboratory markers. These results could suggest that age, educational interventions, and optimized kidney disease management might play a role in promoting cognition health and risk management in this population. Interestingly, the blood pressure of MHD patients showed a significant relationship with their MMSE scores in the univariate analysis, consistent with the findings of Dong et al. Their study indicated that greater variability in home systolic blood pressure was associated with a higher risk of CI in MHD patients. Other contributing factors included age, fewer years of education, interdialytic hypotension, and less frequent hemodialysis sessions [27]. Biochemical markers such as albumin, hemoglobin, and uric acid levels also acted as significant influencing factors. Lower albumin levels and hemoglobin levels were linked to higher CI occurrence, suggesting that malnutrition and anemia, common among MHD patients, might contribute to poor cognitive function. Additionally, elevated uric acid levels, which had been implicated in oxidative stress and neuroinflammation, were associated with higher hemoglobin groups, potentially contributing to better cognitive outcomes [28]. The negative correlation between uric acid and CI suggested potential protective roles of uric acid in cognitive health, aligning with results from the previous studies by Al-Khateeb et al. and Beydoun et al. [29, 30]. These findings emphasize the multifactorial nature of cognitive decline and suggest potential targets for clinical interventions to mitigate cognitive deterioration.

The results of this study underscore the significant and nonlinear relationship between anemia severity and CI in chronic dialysis patients, highlighting the critical role of hemoglobin levels in maintaining health cognitive function. The Q1 and Q2 groups showed an increased risk of CI events, while the Q4 group showed attenuation after adjusting for various confounders, suggesting a potential protective effect of higher adequate hemoglobin levels. Noteworthy, after adjusting for age, sex, education, and dialysis duration, the significant reduction in the OR for Q4 in Model 4 was observed compared to the OR values in Models 1–3. It may be attributed to several potential factors: (1) Multifactorial nature of CI: CI in MHD patients is influenced by numerous factors beyond hemoglobin levels, such as vascular health, inflammatory processes, and metabolic abnormalities. The adjustment for these confounders may have captured these additional contributing factors, which led to a reduction in the apparent effect of hemoglobin on CI in the highest quartile. (2) Complex interactions among covariates: The interaction between age, sex, education, and dialysis duration can have affected the results. For instance, older patients may have more pronounced cognitive decline regardless of their hemoglobin levels, or patients with a longer duration of dialysis may exhibit other factors that contribute to CI, diminishing the observed effect of hemoglobin in this group. (3) Outlier effect: The Q4 group, which consists of patients with hemoglobin levels greater than 130 g/L, may represent a more heterogeneous population with varying underlying conditions. This diversity in patient characteristics can potentially explain the marked decrease in the OR after adjustments for the additional covariates. (4) Dialysis duration and its role in cognitive decline: The duration of hemodialysis can have a particularly strong influence on cognitive outcomes. Longer dialysis duration is often associated with higher levels of uremic toxins, which can negatively impact cognitive function, possibly overshadowing the beneficial effect of higher hemoglobin levels in the Q4 group. Furthermore, anemia can affect cognitive function through several mechanisms. First, it reduces oxygen supply to the brain, which is highly sensitive to oxygen levels, impairing brain metabolism. Second, anemia may increase oxidative stress and inflammation, which can harm brain tissue and lead to cognitive decline. Additionally, anemia can affect brain blood flow, reducing oxygen and nutrient delivery. These factors together may explain the association between anemia and CI, although more research is needed to fully understand the pathways. Xin et al. also concluded that in MHD patients without a history of stroke, there was a high incidence of CI, particularly affecting attention and executive function. Hemoglobin levels may offer protective benefits for cognitive health, while factors such as diabetes, iPTH, and uric acid levels may pose risks. Additionally, depressive and anxiety disorders could exacerbate CI in MHD patients [31]. In Hou′s study, it was demonstrated that anemia was associated with neuron function in CKD patients, and hemoglobin concentration was negatively correlated with striatum function in ESRD patients [8]. Mazumder et al. identified mitochondrial dysfunction and oxidative stress as key molecular mechanisms underlying CI in an adenine-induced CKD animal model [32]. Liu et al. investigated the relationship between spontaneous brain activity and CI by using resting-state functional magnetic resonance imaging (rs-fMRI) method. They revealed that patients with MHD-related CI (MHD-CI) experienced more severe anemia and higher urea nitrogen levels compared to those with MHD without CI (MHD-NCI), with no significant differences in other clinical indicators. A positive correlation between hemoglobin levels and MoCA scores was found, indicating that lower hemoglobin levels were associated with more severe cognitive dysfunction [33]. Reza-Zaldivar et al. found improvements in motor and cognitive functions in rats with CKD treated with recombinant human erythropoietin [34]. Overall, these findings further reinforce the importance of managing anemia in MHD patients, particularly those with severe anemia, and the need for clinical interventions to improve cognitive function. Noteworthy, the relationship between anemia and CI goes both ways. Patients with CI may have difficulties with memory, decision-making, or other cognitive functions, which can lead to poor adherence to medication regimens or dietary recommendations. For instance, patients may forget to take iron supplements or other medications for anemia, which can exacerbate their symptoms. Additionally, CI can also reduce the ability to manage self-care, leading to inadequate nutrition and, consequently, a higher risk of developing anemia. Therefore, the reciprocal relationship between CI and anemia warrants further attention and research.

However, there are controversial research conclusions existed in the relationship between anemia and CI for CKD patients. Tamura et al. reported that in older adults with CKD, anemia did not show an independent link to baseline cognitive function or its decline after controlling for demographic and clinical characteristics. Several possible explanations might be ascribed to it, including the short follow-up period of CKD patients, nutritional deficiencies (vitamin B12, folate, and iron stores), and erythropoietin deficiency [35]. Therefore, the innovative aspects of this study may be summarized as follows: (1) Unlike previous studies, our research focused on the MHD patients in the East China region and we subdivided anemia into four quartiles and analyzed the gradient relationship with cognitive function (MMSE scores), revealing that hemoglobin levels below 110 g/L were an independent predictor of cognitive dysfunction. (2) We explored the effect of different variables on the subcategories of MMSE score across all domains and how comorbidities (e.g., diabetes, cardiovascular disease) affect the anemia-cognitive dysfunction relationship, showing that anemia′s impact remains significant even after adjusting for these factors. (3) Our study highlights that adequate hemoglobin levels independently protect against cognitive dysfunction, offering new evidence for optimizing anemia management in dialysis patients to improve cognitive health. Our findings suggest that anemia can be independently related to CI for MHD patients; however, additional research connecting these potential risk factors with pathological features like neurofibrillary tangles or amyloid plaques is needed to clarify the mechanisms behind CI in patients with ESRD/CKD.

The current study has several limitations: (1) This study is a cross-sectional study and cannot infer the causal relationship between anemia and CI. Further longitudinal studies are needed to establish causality and explore the long-term effects of anemia on cognitive function. However, anemia may still serve as a potential clinical marker for early detection and intervention in cognitive dysfunction. (2) The relatively small sample size of patients with MHD introduces the possibility of selection bias, which can affect the results. (3) Being a single-center study limits the generalizability of the findings and may impact the ability to draw comprehensive conclusions about the relationship between anemia and cognitive dysfunction in MHD patients. (4) This study utilized the MMSE to assess CI. However, the MoCA is more effective in detecting mild CI; therefore, the use of the MMSE may have led to an underestimation of the incidence of CI among patients undergoing MHD. (5) This study did not thoroughly investigate the underlying pathological mechanisms by which anemia might contribute to CI in MHD patients. Additionally, this research focused solely on the correlation between hemoglobin levels and CI in MHD patients, without analyzing the severity or types of CI. To address these issues, future research should consider larger sample sizes and multi-center designs to provide a more detailed understanding of the potential mechanisms between hemoglobin levels and the severity and types of CI.

## Conclusions

In conclusion, this study identifies a significant association between anemia and CI in MHD patients, which emphasizes the importance of monitoring and managing hemoglobin levels in patients with CKD undergoing hemodialysis. Maintaining adequate hemoglobin levels may not only improve physical health but also enhance cognitive function, which is crucial for the overall well-being of this patient population. Therefore, these results provide clinical evidence for understanding the relationship between anemia and CI of MHD patients in Chinese and will be useful for international comparisons. However, further studies are needed to explore the underlying mechanisms linking hemoglobin levels to cognitive function and to identify potential therapeutic strategies for improving cognitive outcomes in these patients.

## Data Availability

The datasets used and analyzed during this study are available from the corresponding author upon reasonable request.

## Abbreviations

CI: Cognitive impairment
MHD: Maintenance hemodialysis
CKD: Chronic kidney disease
ESRD: End-stage renal disease
MMSE: Mini-Mental State Examination test
MoCA: Montreal Cognitive Assessment
Pre-SBP: Pre-dialysis systolic blood pressure
Pre-DBP: Pre-dialysis diastolic blood pressure
25(OH)D: 25-Hydroxyvitamin D
HDL-C: High-density lipoprotein cholesterol
LDL-C: Low-density lipoprotein cholesterol
iPTH: Intact parathyroid hormone
